# Transcriptional regulatory cascade of *LcMYB71* and *LcNAC73* affects low-temperature and drought stress response in *Lonicera caerulea*

**DOI:** 10.3389/fpls.2023.1288947

**Published:** 2023-11-27

**Authors:** Dandan Zang, Yan Sun, Hengtian Zhao

**Affiliations:** Key Laboratory of Mollisols Agroecology, Northeast Institute of Geography and Agroecology, Chinese Academy of Sciences, Harbin, China

**Keywords:** *Lonicera caerulea*, low-temperature and drought stress tolerance, transcriptional regulatory cascade, *LcMYB71*-*LcNAC73*, transient genetic transformation, stable transgenic system

## Abstract

The development of stress tolerance is regulated via the transcriptional regulatory networks involving regulatory homeostasis mediated by protein–DNA interactions. *LcNAC73* from *Lonicera caerulea* was characterized to understand the underlying mechanism of low-temperature and drought stress response in *L. caerulea*. To better understand the transcription pathway of *LcNAC73*, we cloned the promoter and screened proteins that could interact with the promoter. Using Yeast one-hybrid, electrophoretic mobility shift, and chromatin immunoprecipitation assays, we found that the LcMYB71 protein specifically bound to the promoter of *LcNAC73*. The transient transformation and stable transgenic system were used to produce transgenic *L. caerulea* plants with overexpressed and silenced *LcNAC73*, elucidating the effect of *LcNAC73* on low-temperature and drought stress tolerance. *LcNAC73* positively regulated the proline content and enhanced the scavenging of reactive oxygen species, thus improving tolerance to low-temperature and drought stress. Further studies revealed that *LcMYB71* and *LcNAC73* had similar functions and could improve plant low-temperature and drought tolerance. It is necessary to identify the upstream regulators of a specific gene to characterize gene functions and the associated transcriptional pathways.

## 1 Introduction

Abiotic stress is an adverse environmental factor that must be addressed during plant growth and development. Examples of adverse environmental conditions include drought, salinity changes, extreme temperatures, herbivory, and pathogen infections (Liu et al., 2010; Zhu, 2016). Low-temperature stress (LTS) and drought stress are the two main environmental factors that inhibit plant development and have a significant effect on plant distribution and crop production. LTS can cause various unfavorable changes in plant growth and physiology by directly suppressing metabolism or indirectly causing oxidative/osmotic stress (Zhou et al., 2011). Drought is frequently associated with phytohormone production and/or mobilization. Under LTS and drought stress, plants accumulate significant levels of reactive oxygen species (ROS), malondialdehyde (MDA), and other substances. These harmful substances cause oxidative damage to cells, affecting plant growth, development, quality, yield, and survival (Hasanuzzaman et al., 2013). Improving plant tolerance to higher levels of environmental stress is the most effective and direct way of dealing with abiotic stress.

Several biological processes, including cell division, growth, metabolism, and responses to unfavorable environmental conditions, depend on the regulation of transcription (Abdallah and Bauer, 2016). Gene expression is influenced by TFs which bind to the gene promoters. Thus, determining the upstream regulators of genes is necessary to characterize gene expression, identify the associated pathways, and elucidate gene function (Yang et al., 2012).

NAC (NAM, ATAF, and CUC) TFs specific to plants have been linked to various biological activities (Hu et al., 2010). NACs were named after three protein abbreviations: no apical meristem (NAM), *Arabidopsis* transcription activation factor (ATAF1-2), and cup-shaped cotyledon (CUC2) (Aida et al., 1997). The NAC domain consists of approximately 150 amino acids. NACs have a conserved N-terminus related to DNA binding and a highly variable C-terminus related to transcription activation. The number and function of NACs vary across plant genomes (Ooka et al., 2003; Olsen et al., 2005; T et al., 2011; Kumar et al., 2013; Liu et al., 2019). Plant growth (Wu et al., 2015), fruit maturation (Guo and Gan, 2006), leaf senescence (Shan et al., 2012), hormone signaling (Bu et al., 2008), biotic stress, and abiotic stress can all be regulated by NAC TFs (Wang and Dane, 2013). NACs have the potential to improve plant stress tolerance (Wang and Dane, 2013). The plant NAC transcription factor family includes the gene encoding the secondary cell wall NAC domain (SND). However, the physiology of stress tolerance and the molecular mechanism of secondary cell wall-related genes in non-model plants are unknown.

Blue honeysuckle, haskap, sweet berry, and honeyberry are all common names for *L. caerulea* (Rupasinghe et al., 2012). It is primarily found in cold regions of Japan, Eurasia, the Greater Khingan Mountains region of China, and North America (Miyashita et al., 2011; Zhang et al., 2019). This edible berry-bearing plant is extremely hardy and can endure temperatures as low as –46°C without soil cover for safe overwintering (Celli et al., 2014). Additionally, *L. caerulea* is resistant to biological and abiotic stresses such as insects, diseases, and severe drought (Becker et al., 2017). To regulate low-temperature and drought tolerance, it is necessary to study the gene function and regulation mechanism of *L. caerulea* at the molecular level.

In *L. caerulea*, there is no stable genetic transformation system. In this study, transient genetic transformation technology was applied in *L. caerulea* for the first time to determine gene functions. Plant transient transformation systems are widely used in physiological and biochemical analyses and location research (Shan et al., 2019; Jia et al., 2022). The Transient transformation system has several advantages: Gene expression can be studied quickly without compromising the host genome’s stability (Ji et al., 2014). Furthermore, the transient transformation system can help complete genetic transformation in a short period (Zheng et al., 2012). These benefits make transient transformation technology an effective research tool, particularly for plant species lacking a stable genetic transformation system, so the technology is widely used to study gene function (Zang et al., 2017).

**Figure 1:**
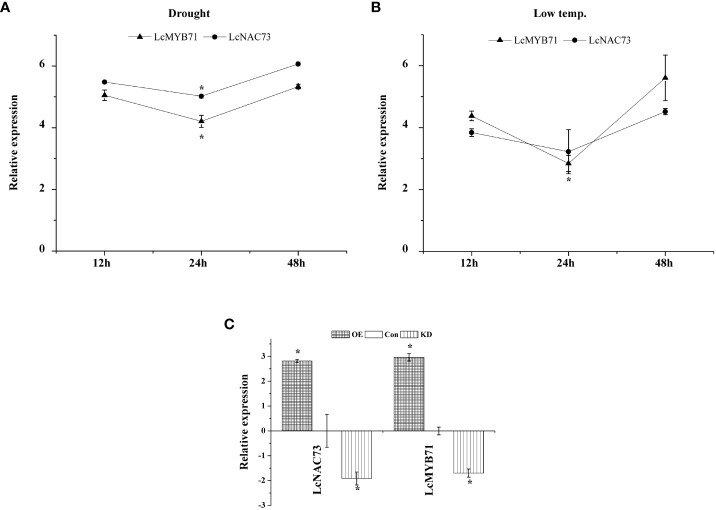
The expression pattern of *LcMYB71* and *LcNAC73* in response to low-temperature or drought treatment. Three independent biological replications were performed. **(A)** The expression pattern of *LcMYB71* and *LcNAC73* in response to drought treatment. All the expression ratios were log2 transformed. *The significant difference (t-test, P < 0.05) compared with treatment for 12h. **(B)** The expression pattern of *LcMYB71* and *LcNAC73* in response to low-temperature treatment. All the expression ratios were log2 transformed. *The significant difference (t-test, P < 0.05) compared with treatment for 12h. **(C)** Analysis of the expression of *LcNAC73* in plants overexpressing or RNAi-silenced for *LcMYB71*. qRT-PCR was performed to analyze the expression of *LcNAC73*. The expression of *LcMYB71* was normalized by that in Con plants and was log2 transformed. *The significant difference (t-test, P < 0.05) compared with Con. OX: *LcMYB71* transformed into *L. caerulea* for transient overexpression; Con: *L. caerulea* transiently transformed with empty pROK2 as a control; KD: pFGC : MYB71 transformed into *L. caerulea* for RNAi-silencing of *LcMYB71*.

We are interested in how stress tolerance conferred by the SND gene to *L. caerulea* develops because *L. caerulea* has strong cold tolerance. First, we cloned two SND genes (GenBank number: OQ024230 and OP117114). We then discovered that SND genes were differentially expressed under LTS (**Figure 1A** and **Supplementary Figure 1**), with *LcNAC73* having the highest level of expression (**Figure 1A**). Therefore, we investigated whether the *LcNAC73* gene possesses low-temperature tolerance. In addition, we speculated that *LcNAC73* may also be involved in other plant stress resistance processes, such as drought stress. Through qRT-PCR analysis, it was found that *LcNAC73* could be induced by drought stress. Therefore, *LcNAC73* was chosen as the target gene in this study to further investigate its function related to tolerance to LTS and drought stress. In addition, determining the upstream regulatory factors of specific genes is of great significance for studying their regulation mechanisms and regulatory networks. The upstream regulatory factors of *LcNAC73* remain unclear. This study reveals a new transcriptional cascade in response to abiotic stress through a series of molecular biological experiments.

## 2 Materials and methods

### 2.1 Plant samples and processing

*Lonicera caerulea* seedlings were grown in pots containing turf peat and vermiculite in a ratio of 2:1 (v/v) in a greenhouse at 24°C, with a relative humidity (RH) of 70%–75% and a photoperiod of 14-h/10-h light/dark. Two months later, the seedlings of *L. caerulea* planted in a normal environment were irrigated using 20% PEG6000 solution for 0, 12, 24, and 48 h.

In the drought simulation test, plant seedlings were grown in the medium. A layer of dialysis membrane MD44 (3500D) (Solarbio, China, Beijing) was wrapped around the medium, and the bottom of the dialysis membrane was sealed with clips (**Supplementary Figure 3A**). The above experimental materials were placed vertically in a flowerpot with a water absorption hole below, and the flowerpot was filled with the medium. The flowerpot was soaked in a 20% PEG6000 solution, and then the solution was poured out of the dialysis bag (**Supplementary Figure 3B**). In the transient transformation test, the lower end of the dialysis membrane was sealed during drought treatment and then sterilized. The sterile operation platform was filled with a sterilized MS solid culture medium. The MS+20% PEG6000 liquid culture medium was added outside the culture medium after it had solidified and the temperature had dropped to room temperature. After soaking for 24 h in MS+20% PEG6000, the solid culture medium containing (Murashige and Skoog) MS was used for subsequent experiments. The cultured seedlings of *L. caerulea* infected with *A. tumefaciens* were placed on a solid medium and grew upwardly (**Supplementary Figures 3C, D**). Subsequently, the 20% PEG6000 stress time was determined. The seedlings were further treated in the same order for 0 h, 12 h, 24 h, and 48 h.

The seedlings were kept at 4°C for 0, 3, 6, 12, and 24 h. Then, whole plants were collected at specific time points to conduct subsequent analyses. At least 10 seedlings were collected for the analysis in each biological replicate, and three independent biological replicates were used.

The *Arabidopsis thaliana* WT plant seeds were from ABRC (CS70000), and *Lonicera caerulea* seeds were obtained from the Northeast Institute of Geography and Agroecology of Chinese Academy of Sciences in China.

### 2.2 Cloning the *LcNAC73* promoter

Based on the *LcNAC73* sequence generated using the genome walking kit (Takara, Dalian, China), thermal asymmetric interlaced PCR (TAIL-PCR) was performed to amplify the promoter. The primers used for TAIL-PCR are provided in **Supplementary Table 1**. The programs PlantCARE (http://bioinformatics.psb.ugent.be/webtools/plantcare/htmL/) and PLACE (http://www.dna.affrc.go.jp/PLACE) were used to predict the cis-acting motifs in the *LcNAC73* promoter.

### 2.3 Vector establishment and transient genetic transformation

The promoter was fused with the firefly luciferase (LUC) reporter gene using the plasmid pGreen II0800-LUC. The *LcNAC73* promoter fragments were 1869, 1296, 771, and 363 bp long (representing the length from the start point of the ATG translation sequence to the start of the upstream fragment). The promoters of pro1 (p1) and pro2 (p2) were inserted into pGreen II0800-LUC, as previously described. All fragments were cloned from the *L. caerulea* DNA, and reporter vectors were generated. The pGreen II0800-LUC vector was used as the negative control (NC). Each construct was later subjected to electroporation to the *A. tumefaciens* strain EHA105 and transformation to *N. benthamiana* using the transient expression assay method (Bian et al., 2020). A living fluorescence imager (DynaPlant Desktop, Microlens Technologies, Beijing, China) was used to test luciferase signaling. For the infiltrated *N. benthamiana* leaves, the dual-luciferase reporter gene assay kit (Beyotime, Shanghai, China) was used to determine the transcriptional activity, which was expressed as the LUC-to-REN ratio. All experiments were performed in three independent biological replicates.

Later, the coding sequences (CDSs) of *LcNAC73* and *LcMYB71* were fused with pROK2, which was controlled by the CaMV 35S promoter, to construct the plant overexpression vector. The 257-bp truncated cDNA sequence of *LcNAC73* was inserted into the RNAi vector pFGC5941, and *LcNAC73* was silenced by inserting inverted repeats at bilateral CHSA intron ends (pFGC: NAC73). Then, *L. caerulea* was genetically transformed with pROK2-*LcNAC73* (for overexpressing *LcNAC73*, OX-NAC73), pROK2 (control, Con), and pFGC : NAC (for silencing *LcNAC73*, KD-NAC73). Similarly, a 246-bp truncated cDNA sequence was inserted into the RNAi vector pFGC5941, and *LcMYB71* was silenced by inserting inverted repeats at the ends of the bilateral CHSA introns (pFGC: MYB71). pROK2-MYB71 (for overexpressing *LcMYB71*, OX-MYB71), pROK2 (Con), and pFGC : MYB (for silencing *LcMYB71*, KD-MYB71) were then used to genetically transform *L. caerulea*. The information on all primers is presented in **Supplementary Table 1**.

The method described by Ji et al. (Ji et al., 2014) was used for transformation, with slight modifications. Briefly, a liquid lysogeny broth (LB) medium containing rifampicin (50 mg/L) and kanamycin (50 mg/L) was used to grow vector-transfected *A. tumefaciens* EHA105 colonies. This was followed by culturing at 28°C with shaking at 180 rpm until the absorbance (OD) reached 0.6–0.7 at 600 nm (OD_600_). Then, the cultures were centrifuged at 3000×g for 10 min, followed by adjustment of the cells to an OD_600_ of 0.8 using the transformation solution [MS + 2.5% (w/v) sucrose + 100 µM acetosyringone + 0.01% (v/v) Tween20, pH 5.8]. To conduct transient genetic transformation, each plant was immersed in the transformation solution for 7 h at 25°C with shaking at 100 rpm. The plant was then rinsed immediately using distilled water to remove excess *A. tumefaciens* cells, and the samples were covered with sterile filter paper to remove excess water. The plant samples were then grown vertically in the solid medium [MS + 1% (w/v) sucrose + 100 µM acetosyringone + 4.44 µM BA + 4.92 µM IBA, pH 5.8] for 48–72 h. At 48 h after culture, the transformation was completed, and each plant was used for further analysis, including LTS or 20% PEG6000 treatment (**Supplementary Figures 3E–H**).

To validate the transient transformation efficiency, the pCAMBIA1301 vector was transformed into *L. caerulea* using our transient transformation system, and GUS staining and GUS activity of *L. caerulea* were analyzed after 2 h, 4 h, and 7 h of transformation. The method described by Blázquez was used to determine GUS activity (Blázquez, 2007). All the experiments were performed in three independent biological replicates.

### 2.4 Determination of the upstream regulator of the *LcNAC73* gene using Y1H analysis

To identify the TFs that bound to the promoter of *LcNAC73*, Y1H assays were performed. We constructed a yeast one-hybrid library containing four MYB genes (GenBank numbers: OP117115, OQ145321, OQ145322, OQ145323) using the SMART^®^ cDNA Library Construction Kit (Takara Bio, USA). The pHIS2 vector (Takara) was then cloned with different sites of the *LcNAC73* promoter to form the bait (pHIS2-pro1 to 5, **Supplementary Table 1**). A Y1H assay was performed to screen the prey cDNA library to investigate protein-truncated promoter interactions.

### 2.5 Analysis of subcellular localization

To construct the plant expression vector 35S: NAC73-GFP, *LcNAC73* CDS without any termination codon was fused with green fluorescent protein (GFP) CDS at its 5′-end under the control of the CaMV 35S promoter. The GFP CDS regulated by the CaMV 35S promoter (35S:GFP) served as the reference, whereas the vector pCAMBIA1300-mKate-NLS (Shcherbo et al., 2007) was used as the positive nuclear control. The 35S:GFP and 35S: NAC73-GFP controls were then transfected into *Arabidopsis* protoplast cells (Zhang et al., 2009). A confocal laser scanning microscope (C2-ER; Nikon) was used to visualize the transformed *Arabidopsis* protoplast cells.

### 2.6 Chromatin immunoprecipitation (ChIP) assay

The Chromatin immunoprecipitation (ChIP) assay was performed to investigate the binding of LcMYB71 to the *LcNAC73* promoter. After the transient transformation of 35S:MYB71-GFP in *L. caerulea* plants, a ChIP assay was performed using the transformed *L. caerulea* plants following the method described by Zang et al. (Zang et al., 2017) with some modifications. Briefly, the protein was cross-linked with chromatin DNA using 1% formaldehyde, with one-third of chromatin as the input reference. The chromatin was then sheared into 0.3–0.8 kb fragments using ultrasound and divided into two parts, with one part subjected to immunoprecipitation using the anti-GFP antibody (ChIP+) and the other part using the rabbit anti-hemagglutinin (HA) antibody for NC (ChIP−). The DNA fragments of the immunoprecipitated complexes were released by incubation for 6 h at 65°C. Chloroform was then added to extract and purify the immunoprecipitated DNA, followed by PCR and gel electrophoresis for product visualization. PCR was performed under the following conditions: initial denaturation at 94°C for 3 min, followed by 35 cycles of 94°C for 30 s, 58°C for 30 s, and 72°C for 30 s, and finally 7 min at 72°C. The primers used for PCR are listed in **Supplementary Table 2**. An Agilent AriaMx (Agilent Stratagene, USA) was used for ChIP-qPCR. The PCR was carried out as follows: initial denaturation at 94°C for 30 s, followed by 40 cycles of 94°C for 12 s, 60°C for 30 s, 72°C for 40 s, and 82°C for 1 s. The endogenous control was the *LcTUB1* sequence (GenBank number: MT344114).

### 2.7 Modulation of *LcNAC73* via *LcMYB71*

To determine the role of *LcMYB71* in regulating the expression of *LcNAC73*, various truncated promoters of *LcNAC73* were fused using pGreen II0800-LUC to drive the expression of LUC genes as reporters (**Supplementary Table 1**). The construct 35S:*LcMYB71* served as an effector. The transformation of the reporter construct and effector construct 35S:*LcMYB71* was then completed in *N. benthamiana* through transient genetic transformation. Finally, the effector was transformed into *N. benthamiana* as a NC.

### 2.8 Physiological and phenotypic analysis

Nitroblue tetrazolium (NBT) and 3,3′-diaminobenzidine (DAB) were used for the histochemical staining of O_2_^−^ and H_2_O_2_. Membrane injury was detected by Evans blue staining, following the method described by Romero-Puertas et al. (Romero-Puertas et al., 2004). Forty-eight hours after transient genetic transformation, the OE, KD, and Con *L.caerulea* plants were irrigated with 20% PEG6000 solution or treated at 4°C. The chlorophyll content was then measured according to the method described by Lichtenthaler (Lichtenthaler, 1987), while the SOD/POD activities and electrolyte leakage rate (ELR) were measured according to the method described by Verma et al. (Verma and Mishra, 2005). The proline content was determined using the method described by Ábrahám et al. (Ábrahám et al., 2010). MDA levels were determined using a Plant Malondialdehyde (MDA) assay kit (colorimetric method) (Jiancheng, Nanjing, China). ROS levels were measured using the ELISA method with a ROS ElISA Kit (Sbjbio, Nanjing, China). At least 10 seedlings were collected for the analysis in each biological replicate, and three independent biological replicates were used. The 4-week-old WT, OE4, OE10, and OE11 lines were irrigated again for 3 days after stopping irrigation for 16 days, and then the plant phenotypes were observed. Four-week-old WT, OE4, OE10, and OE11 lines were exposed to LTS at -20°C for 15 min and then allowed to grow under normal conditions, and the plant phenotypes were observed 7 days later.

### 2.9 Quantitative real-time PCR (qRT-PCR) assay

The CTAB approach was used to extract the total RNA of *L. caerulea*. Contaminated DNA was removed following digestion with DNase I. cDNA was prepared from 1 µg of total RNA using the Primescript™ RT reagent kit (Takara Bio, USA), with oligo (dT) used as the primers. cDNA was then diluted to 100 µL and used as a template for PCR. Two endogenous controls were used to analyze *LcMYB71* and *LcNAC73* in the *L. caerulea* plants: *LcTUB1* (GenBank number: MT344114) and *LcACT1* (GenBank number: MT344113). To analyze gene expression in the OX-NAC73, KD-NAC73, and Con *L. caerulea* plants, *LcTUB1* and *LcACT1* were used as endogenous controls. PCR was performed on the Agilent AriaMx. A cDNA template (2 µL), primers (1 µL each), the SYBR Green Real-time PCR Master Mix (10 µL, Toyobo, Japan), and ddH_2_O were included in the PCR mixture (20 µL). The following conditions were used for PCR: initial denaturation at 94°C for 30 s, followed by 40 cycles of 94°C for 12 s, 60°C for 30 s, 72°C for 40 s, and 82°C for 1 s. The efficiency of PCR amplification was determined based on the melting curves for all samples. The 2^−ΔΔCt^ approach was used to determine gene expression, and three independent biological replicates were set for each sample (Livak and Schmittgen, 2001). The primers for PCR are presented in **Supplementary Table 2**.

### 2.10 Production of recombinant protein

The CDSs of *LcMYB71* were cloned into the pCZN1 vector (Zoonbio, Nanjing, China) containing the 6×His tag in the N-terminal according to the specific protocols. To produce recombinant proteins, the constructs were transfected into *Escherichia coli* Arctic Express (DE3) (Weidi Biotechnology, Shanghai, China). HisPur Ni-NTA Resin (Thermo Scientific) was used to purify the recombinant protein. The primers used to develop the construct are listed in **Supplementary Table 2**. The pCZN1-*LcMYB71* recombinant proteins were isolated using 12% SDS-PAGE (**Supplementary Figure 6**).

### 2.11 Electrophoretic mobility shift assay (EMSA)

The electrophoretic mobility shift assay (EMSA) was conducted to detect LcMYB71 directly binding to DNA fragments of the *LcNAC73* promoter. The LcMYB71 protein was obtained from *E. coli*, as described previously. The Biotin 3’-End DNA Labeling Kit (Thermo Scientific) was used to label the DNA fragments of the *LcNAC73* promoter with biotin. The primers used to prepare probes are presented in **Supplementary Table 3**. The LightShift Chemiluminescent EMSA Kit (Thermo Scientific) was used for EMSA, following the manufacturer’s instructions. The protein–DNA complexes were separated using 6% (w/v) native PAGE and transferred to a nylon membrane (Thermo Scientific). The signal was detected using chemiluminescence. Student’s t-test was carried out using the SPSS software (v.17.0) for all the statistical analyses to determine significant (*, P < 0.05).

## 3 Results

### 3.1 LcNAC73 cloning and analysis of *L. caerulea*

The full-length cDNA sequence of *LcNAC73* in *L. caerulea* was cloned. The CDS of *LcNAC73* was 729 bp long and encoded 242 aa. The neighbor-joining method was used for phylogenetic analysis using the software Clustal X software (version 1.81). *LcNAC73* was compared with other NAC proteins from *A. thaliana*, *O. sativa*, *Z. mays*, *B. platyphylla*, *V. vinifera*, and *P. trichocarpa*. Phylogenetic tree analysis revealed that *LcNAC73* was a member of the NAC family (**Supplementary Figures 4A, B**), and its homologous protein was NAC73, so it was designated as *LcNAC73*.

### 3.2 *LcNAC73* is a nuclear protein

**Figure 2:**
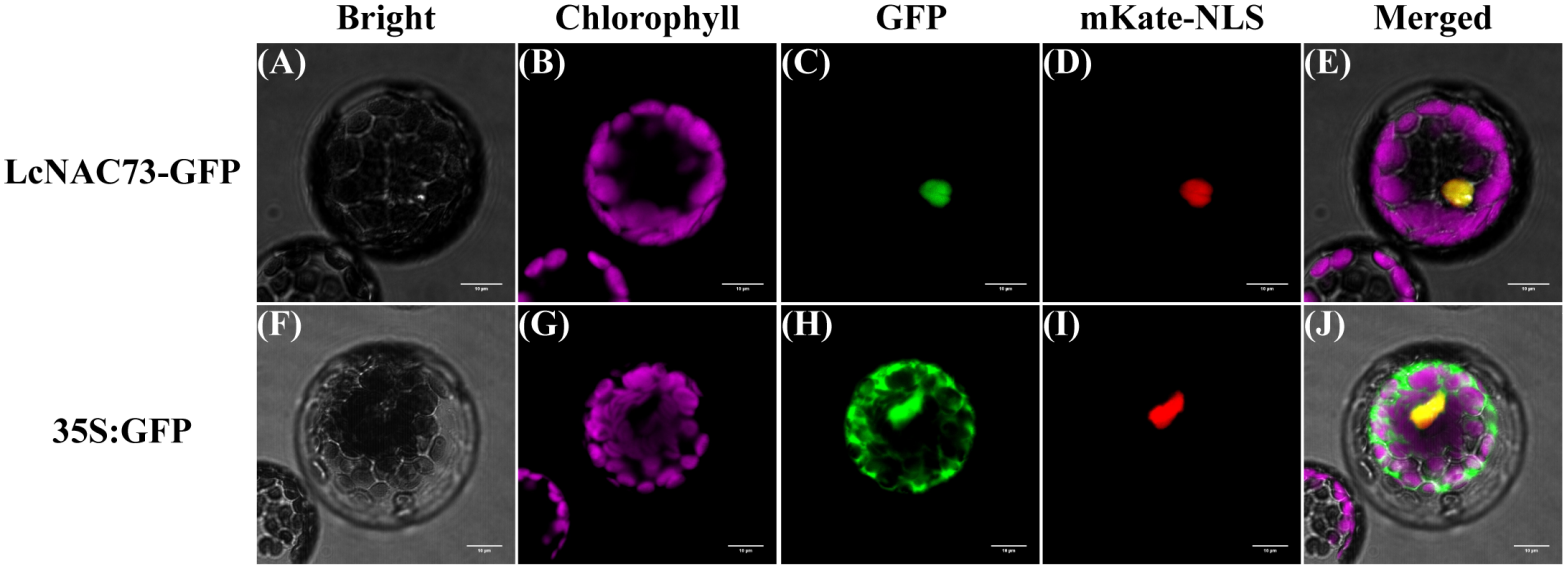
Subcellular localization of LcNAC73 protein. Microscopic analysis of LcNAC73 within the protoplasts of *A. thaliana*. The vectors (pCAMBIA1300-mKate-NLS and 35S:LcNAC73-GFP) **(A–E)** and control vectors **(F–J)** (pCAMBIA1300-mKate-NLS and 35S:GFP) were transferred into mesophyll protoplasts of *A. thaliana*, respectively. Cells were observed by confocal microscopy and three independent biological replications were performed. Scale bars = 10 µm.

We fused the green fluorescent protein (GFP) to C-terminal of LcNAC73 to determine the subcellular localization experimentally. After co-transfection of pCAMBIA1300-mKate-NLS vector and recombinant *LcNAC73*-GFP vector into *Arabidopsis*, mKate and GFP fluorescence signals were observed in the nucleus. The results indicated that 35S:*LcNAC73*-GFP was only detected in the nucleus (**Figures 2A–E**). However, the nuclear and cytoplasmic fluorescent signals from 35S: GFP were detected (**Figures 2F–J**), suggesting *LcNAC73* is a nuclear protein.

### 3.3 Generation of transient *LcNAC73*-overexpressed or knockdown *L. caerulea* plants

**Figure 3:**
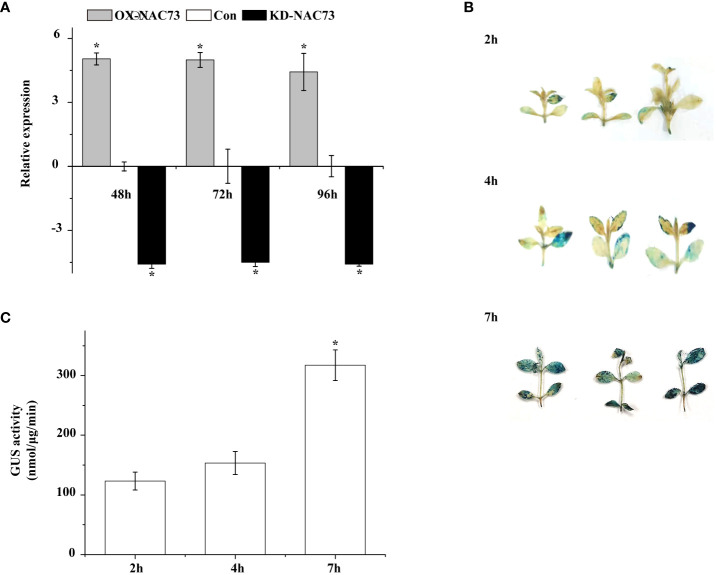
GUS activity and RT-PCR were used to analyze the stability of transient genetic transformation in *L. caerulea* plants. Three independent biological replications were performed. **(A)** The transcript level of *LcNAC73* in OX-NAC73, KD-NAC73, and Con plants under normal conditions. The expression data were log2 transformed. *The significant difference (t-test, P < 0.05) compared with Con. One-month-old *L. caerulea* plants were transiently transformed with empty pROK2, 35S:NAC73 or pFGC5941-NAC73. After transformation for 48 h, *L. caerulea* plants were grown under normal conditions for 48, 72, and 96 h respectively, and the expressions of *LcNAC73* in whole OX-NAC73, KD-NAC73, and Con plants were determined. Con plants were transformed with empty pROK2; OX-NAC73: plants were transformed with 35S:NAC73 for overexpression of *LcNAC73*; KD-NAC73: plants were transformed with pFGC5941-NAC73 for silencing the expression of *LcNAC73*. **(B)** The transient expression of GUS gene in *L. caerulea* plants was analyzed by histochemical method after being infected by *A. tumefaciens* for 2 h, 4 h, and 7 h respectively. **(C)** The GUS activities of *L. caerulea* plants infected by *A. tumefaciens* for 2 h, 4 h, and 7 h were determined by fluorescence analysis. *The significant difference (t-test, P < 0.05) compared with treatment for 2h.

Three *L. caerulea* plants with transient transformation were obtained in this study, namely plants with transient *LcNAC73* overexpression (OX, under 35S:NAC transfection), control (Con, under blank pROK2 transfection), and transient *LcNAC73* knockdown (KD, under pFGC : NAC transfection). To determine the expression of *LcNAC73* in OX, Con, and KD plants, qRT-PCR was performed. After transfection for 48 h, the plants were subjected to 20% PEG6000 treatment or LTS or were planted in a normal environment for 24 h or 48 h to analyze the expression of *LcNAC73* in plants from the OX, Con, and KD groups. After 24 h, the OX group had a significantly higher expression of *LcNAC73* than did the Con group after LTS and drought stress. In contrast, the KD group had a significantly lower expression levels than did the Con group after LTS and drought stress (**Figure 3**). These findings suggest that transient transformation technology can be used to create plants overexpressing or with knocked down *LcNAC73*. Stress tolerance was evaluated after *LcNAC73* expression in the OX and KD groups stabilized after 24 h (**Figure 3**). These constructed plants could be used for gain and loss-of-function assays.

The transient transformation system was used to transfect pCAMBIA1301 to determine the efficiency of the transient transformation system in *L. caerulea* plants. GUS gene expression was detected in the *L. caerulea* plants after GUS staining and GUS activity (**Figures 3B, C**). Transient transformation technology was effective for gene transformation in *L. caerulea*, and the transformation efficiency reached its best after 7 h.

### 3.4 Physiological analysis of *LcNAC73*

**Figure 4:**
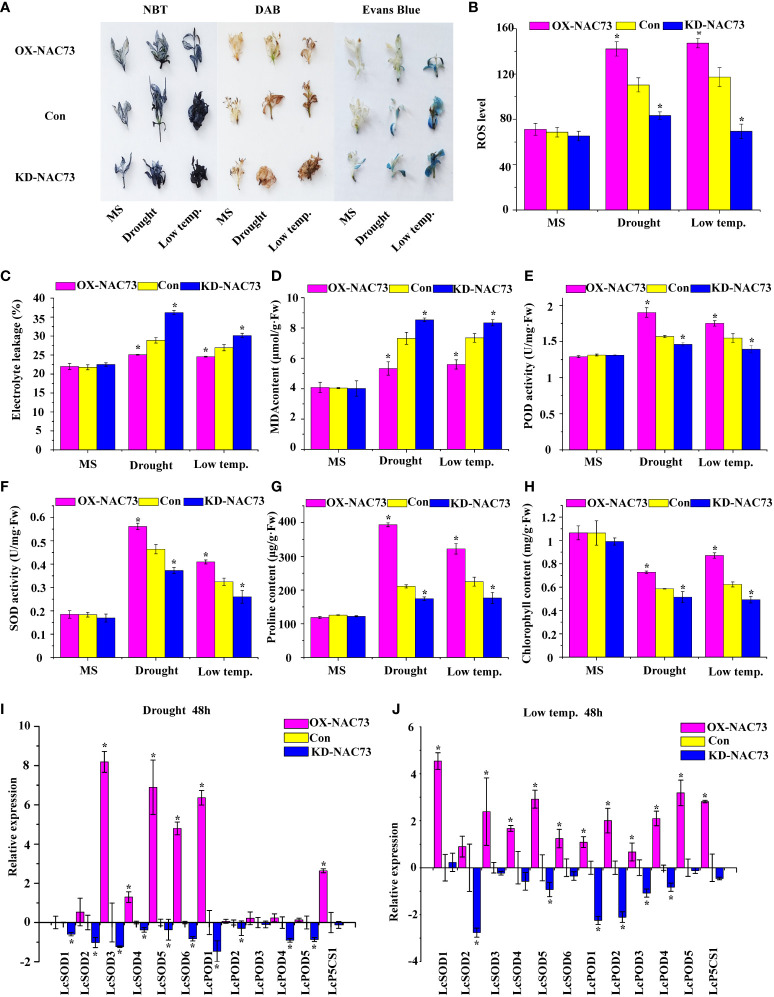
Analysis of the physiological responses mediated by *LcNAC73*. Three independent biological replications were performed. The data represent the mean values of the three independent experiments. Error bars indicate the SD. *The significant difference (t-test, P < 0.05) compared with Con. **(A)** NBT, DAB, and Evans blue staining. **(B)** ROS level. **(C)** Electrolyte leakage rate. **(D)** MDA content analysis. **(E)** POD activity. **(F)** SOD activity. **(G)** proline content. **(H)** chlorophyll content. OX-NAC73: *LcNAC73* transformed into *L. caerulea* for transient overexpression; Con: *L. caerulea* transiently transformed with empty pROK2 as a control; KD-NAC73: pFGC5941-NAC73 transformed into *L. caerulea* for RNAi-silencing of *LcNAC73*. 1/2MS: grown in 1/2MS medium as the normal condition control. **(I, J)** Analysis of the expression of SOD, POD, and P5CS genes in the Con, OX-NAC73, and KD-NAC73 plants under drought stress (20% PEG6000) for 48 h or low-temperature stress (4°C) for 48 h. The GenBank numbers for the studied genes are shown in **Supplementary Table 2**.

Physiological changes associated with stress tolerance to abiotic stress were analyzed to determine the role of *LcNAC73* in mediating stress tolerance to LTS and drought stress. DAB and NBT were also used to stain the two major ROS, O_2_^−^ and H_2_O_2_. Under normal conditions, the O_2_^−^ and H_2_O_2_ levels were stable in the plants. However, under LTS and drought stress, their levels decreased significantly in the OX-NAC73 plants and increased in the KD-NAC73 plants compared with the Con plants (**Figure 4A**). According to the results of Evans blue staining, the staining intensities of the OX-NAC73, KD-NAC73, and Con plants were similar under normal conditions. However, after PEG or LTS treatment, membrane injury was lower in the OX-NAC73 plants and higher in the KD-NAC73 plants than in the Con plants (**Figure 4A**). Furthermore, after treatment with 20% PEG6000 or exposure to LTS, the ELR was significantly lower in the OX-NAC73 plants and significantly higher in the KD-NAC73 plants than in the Con plants (**Figure 4C**). This observation agrees with the results of Evans blue staining (**Figure 4A**). ROS level analysis and histochemical staining showed the same trend (**Figure 4B**). After treatment with 20% PEG6000 or exposure to LTS, the OX-NAC73 plants showed lower MDA levels than did the Con plants. In contrast, the KD-NAC73 plants showed a higher MDA level than did the Con plants (**Figure 4D**). Furthermore, in the OX-NAC73, KD-NAC73 and Con plants, SOD and POD activities and proline content remained stable in a normal environment with little difference. However, under 20% PEG6000 or LTS, these activities or contents increased considerably in the OX-NAC73 plants and decreased in the KD-NAC73 plants compared with those in the Con plants (**Figures 4E–G**). In the OX-NAC73, KD-NAC73, and Con plants, chlorophyll levels remained stable in a normal environment, with little difference. However, after low-temperature or drought treatment, the chlorophyll levels in OX-NAC73 plants decreased the least, followed by the Con plants, and KD-NAC73 plants showed the greatest decrease in chlorophyll levels (**Figure 4H**). Thus, *LcNAC73* enhanced the tolerance to low-temperature or drought stress.

### 3.5 *LcNAC73* modulated the expression of SOD and POD genes and proline biosynthesis

The *L. caerulea* plants overexpressing *LcNAC73* showed enhanced SOD and POD activities, as well as elevated proline levels under low-temperature or 20% PEG6000 stress. Therefore, the genes associated with SOD and POD, activities and proline biosynthesis were investigated under 20% PEG6000 or LTS. The expression of *LcSOD1-6*, *LcPOD1-4*, and *LcP5CS1* in the transient transformation of *L. caerulea* was analyzed. The OX-NAC73 plants expressed a high level of *LcSOD1-6*, *LcPOD1-4*, and *LcP5CS1*, while the KD-NAC73 plants expressed a low level, and the Con plants expressed an intermediate level (**Figures 4I, J**). These findings indicated that *LcNAC73* overexpression increased the expression levels of P5CS, SOD and POD gene, thereby increasing SOD and POD activities and proline content.

### 3.6 *LcNAC73* confers stress tolerance to LTS and drought stress in transgenic *Arabidopsis* plants

**Figure 5:**
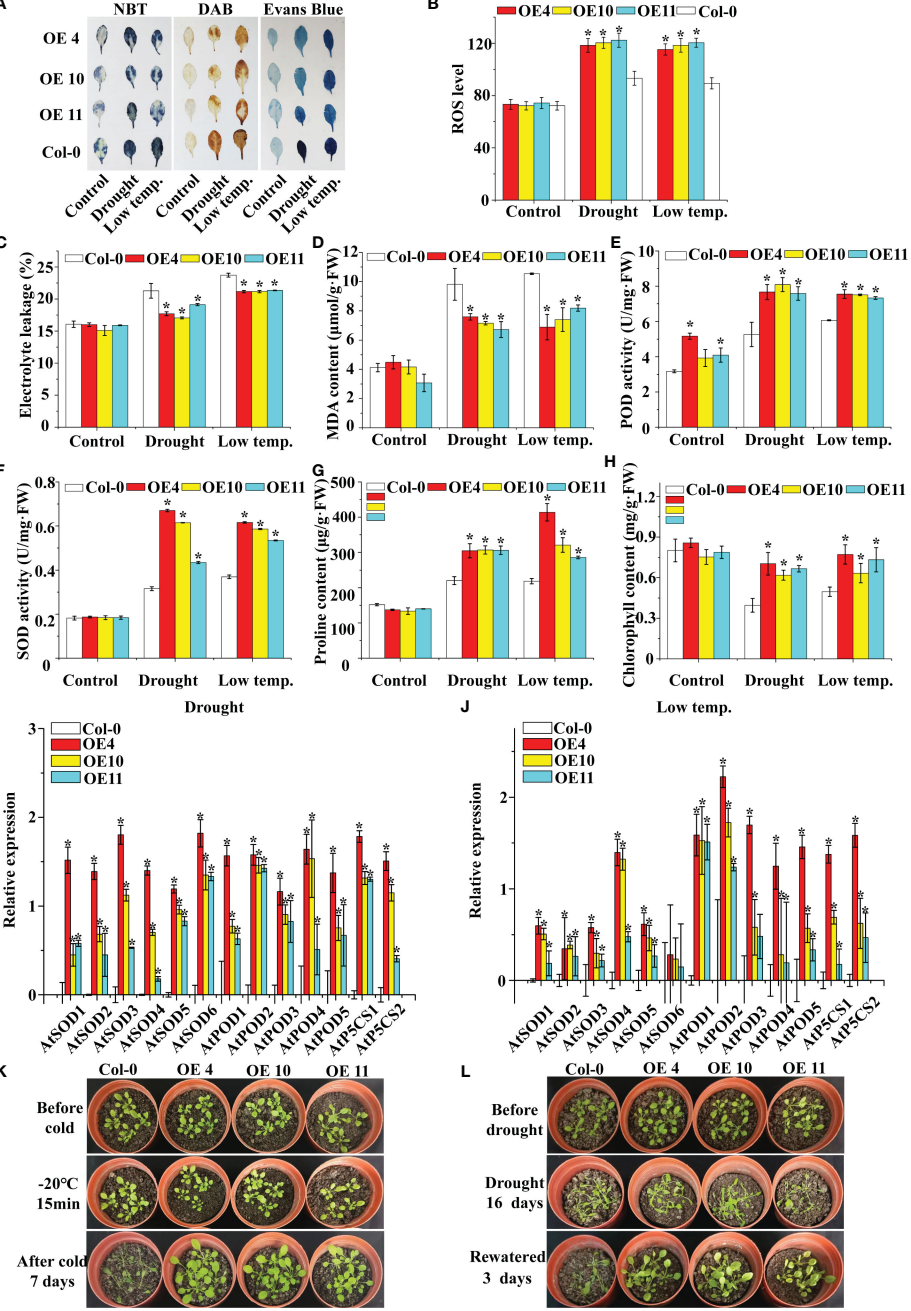
Analysis of ROS scavenging capability and proline content in *LcNAC73* transformed into *Arabidopsis* plants. Three independent biological replications were performed. Error bars indicate the SD. **(A)** The leaves of WT and *LcNAC73* transformed into *Arabidopsis* plants were analyzed by staining with DAB, NBT, and Evans blue; **(B)** ROS level; **(C–H)** Comparison of Electrolyte leakage rate **(C)**, MDA content **(D)**, POD activity **(E)**, SOD activity **(F)**, proline content **(G)**, and chlorophyll content **(H)** between *LcNAC73* transformed lines and WT *Arabidopsis* plants. **(I, J)** The expression levels of SOD, POD, and P5CS genes in WT and *LcNAC73* transformed into *Arabidopsis* plants under normal, low-temperature, or drought treatment conditions. **(K, L)** Phenotypes of *LcNAC73* transformed lines and WT Arabidopsis plants under drought and low temperature stress. The expression level of each gene in transgenic plants was normalized using that in WT plants. The expression data were log2 transformed. *The significant difference (t-test, P < 0.05) compared with WT plants.

To determine the activity of *LcNAC73*, *Arabidopsis* plants overexpressing *LcNAC73* were collected, and 10 T_3_ homozygous lines were acquired. The results of the qRT-PCR assay showed that *LcNAC73* was successfully expressed in each transgenic line (**Supplementary Figure 5**). Three lines (4, 10, and 11) were selected for subsequent analysis. DAB staining indicated that the O_2_^−^ or H_2_O_2_ level in *Arabidopsis* plant leaves remained stable under normal growth conditions (**Figure 5A**). In contrast, under 20% PEG6000 and LTS, WT plants had significantly higher O_2_^−^ and H_2_O_2_ levels than did transgenic plants (**Figure 5A**). Overexpression of *LcNAC73* reduced cell death under LTS and drought stress, according to the results of the ELR and Evans Blue staining (**Figures 5A, C**). The OE plants had lower MDA levels than did the Con plants under low-temperature or drought stress (**Figure 5D**). Similarly, after low-temperature or PEG stress, the plants in the transgenic lines had significantly higher SOD, POD, and ROS activities than did the WT plants (**Figures 5B, E, F**). Furthermore, under LTS and drought stress, the *LcNAC73*-transfected plants had a significantly higher proline content than did the WT lines (**Figure 5G**). Chlorophyll levels in transgenic lines and WT plants remained similar under normal conditions (**Figure 5H**). Under LTS and drought stress, however, chlorophyll levels decreased significantly more in the WT lines than in the transgenic lines (**Figure 5H**), indicating that *LcNAC73* overexpression reduced chlorophyll loss after PEG treatment or low-temperature exposure. *LcNAC73*-overexpressing *Arabidopsis* plants had higher proline content, SOD, and POD activities than did the WT lines. Additionally, the expression levels of P5CS, SOD, and POD genes in WT and transgenic *Arabidopsis* plants were analyzed. P5CS, SOD, and POD genes expression levels were significantly higher in *Arabidopsis* plants transfected with *LcNAC73* under PEG and LTS than did the WT lines (**Figures 5I, J**). It was discovered that the survival rate of the overexpression lines was significantly higher than that of the WT lines by observing the phenotypes of WT, OE4, OE10, and OE11 lines under low-temperature or drought stress (**Figures 5K, L**). These findings demonstrated that *LcNAC73-* overexpressing plants were more tolerant to LTS and drought stress than the WT plants.

### 3.7 Cloning the *LcNAC73* promoter

**Figure 6:**
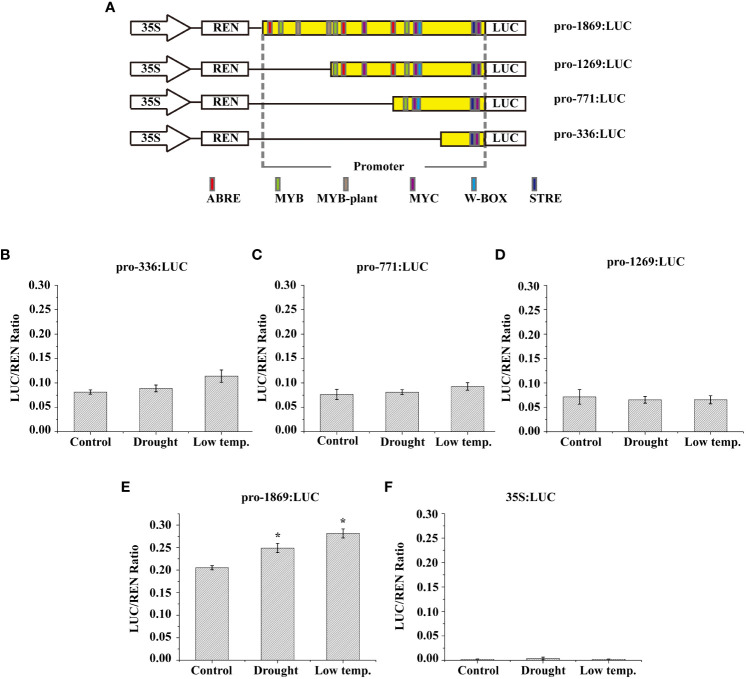
Low-temperature and drought responsiveness of *LcNAC73* promoter. Four independent biological replications were performed. Data are means ± SE. **(A)** The numbers of each construct indicate the distance from the start codon ATG. The cis-elements predicted using the PLACE and Plant CARE databases are represented by their respective labels. **(B–F)** Relative LUC/REN ratio in *L. caerulea* with transient genetic transformation carrying the vectors described above and negative control (35S:LUC) under different treatments. Drought: After transient genetic transformation for 48 h, the *L. caerulea* plants were irrigated with a solution of 20% PEG for 48 h; Cold: After transient genetic transformation for 48 h, the *L. caerulea* plants were treated with 4°C for 48 h; Control: After transient genetic transformation for 48 h, the *L. caerulea* plants were grown without treatment.

TAIL-PCR was conducted to clone an upstream fragment of the transcription start site containing the 729-bp *LcNAC73* promoter sequence. The regulatory motifs of the promoter sequence were predicted, and cis-acting elements, including ABRE, MYC, MYB, and W-box (**Figure 6A**), were identified. This suggests that protein binding to the *LcNAC73* promoter could influence the expression of *LcNAC73*.

The reporter gene was fused with three truncated and full-length fragments of the *LcNAC73* promoter (1,269, 771, 363, and 1869 bp) (**Figure 6A**), followed by transfection in *N. benthamiana* to determine the response region to low-temperature or drought. The pGreen II0800-LUC vector was transfected with NC (**Figure 6F**). In the empty vector, the activity of LUC/REN was absent in the *N. benthamiana* lines or under PEG and LTS. The LUC/REN activity of the truncated fragments of the *LcNAC73* promoter (pro-1269, pro-771, and pro-363) showed little change under PEG and LTS (**Figures 6B–D**). In contrast, the LUC/REN activity of the truncated fragments of the pro-1869 varied significantly (**Figure 6E**).

Based on the above results, we hypothesized that the *LcNAC73* gene was strongly associated with stress tolerance to LTS and drought stress. These results indicate that the promoter of the *LcNAC73* gene is an inducible promoter that responds to different types of stress. In addition, the promoter regions (1269–1869 bp) had strong effects on the response of the plant to low-temperature or drought stress.

### 3.8 Transcriptional activation of *LcNAC73* via the direct combination of *LcMYB71* and the *LcNAC73* promoter

**Figure 7:**
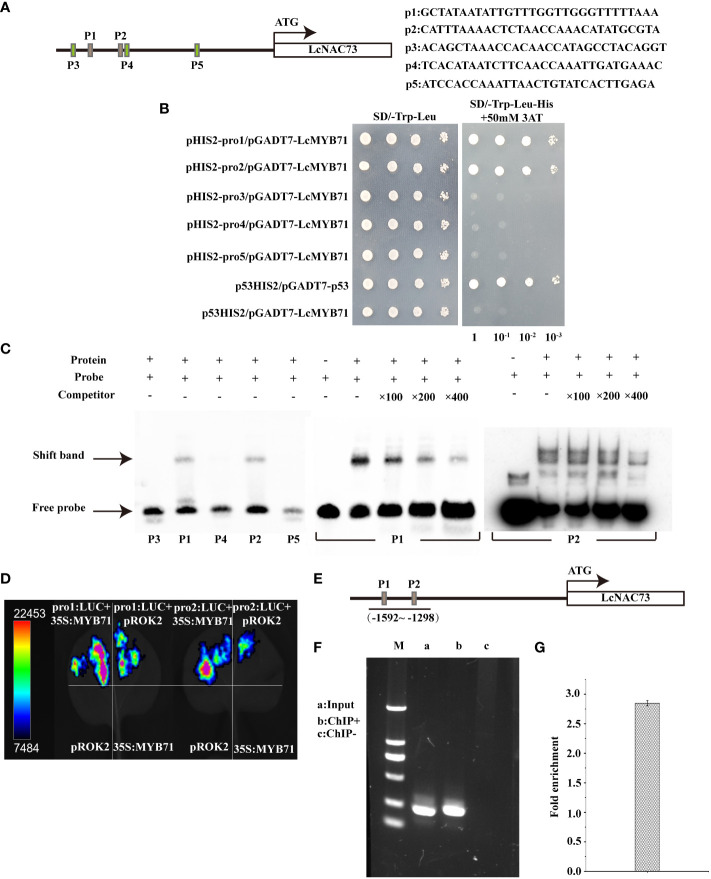
Binding of LcMYB71 to the promoter of *LcNAC73*. Three independent biological replications were performed. **(A)** Schematics diagram of the *LcNAC73* promoter (left) and sequences used in the EMSA (right). **(B)** Analysis of the binding of LcMYB71 to the truncated *LcNAC73* promoter using Y1H. **(C)** EMSA shows the binding of LcMYB71 to the promoter of *LcNAC73*. The “+” and “−” indicate the presence and absence of the indicated probe or protein. 100-, 200-, and 400-fold excess unlabeled probes were used for competition. **(D)** Transient expression assays show that LcMYB71 activates the expression of pro1:Luc, pro2:Luc, pro3:Luc, pro4:Luc, and pro5:Luc. Representative images of *N. benthamiana* leaves 72 h after infiltration were shown. **(E)** Schematics diagram of the *LcNAC73* promoter and the truncated promoter amplified by PCR. **(F, G)** The amplified promoters of *LcNAC73* were visualized by gel electrophoresis **(F)** and ChIP-qPCR analysis of the enrichment of the truncated promoter of *LcNAC73* **(G)**. Input, the sonicated chromatin from 35S:MYB71-GFP; ChIP+: the chromatin immunoprecipitated by an anti-GFP antibody. ChIP−: chromatin immunoprecipitated by an anti-hemagglutinin (HA) antibody.

The 1269 to 1869 bp region in the *LcNAC73* promoter strongly influenced the drought or low-temperature response, where two MYB-plant motifs were detected. A Y1H assay was performed to screen for proteins binding to pro1 (p1) and pro2 (p2). LcMYB71 (GenBank number: OP117115) was the target protein. To determine the role of LcMYB71 in activating gene levels by combining with the promoter of *LcNAC73*, truncated *LcNAC73* promoters containing pro1 (p1), pro2 (p2), pro3 (p3), pro4 (p4), and pro5 (p5) (**Figure 7A**) were inserted into pHIS2. A Y1H assay was conducted to determine the specific binding of LcMYB71 to the promoter. The result showed that LcMYB71 failed to bind to the p3, p4, and p5 regions, indicating that LcMYB71 bound specifically to the p1 and p2 regions (**Figure 7B**).

To determine whether LcMYB71 could bind to the p1 and p2 regions of the *LcNAC73* promoter, we performed the EMSA using p1, p2, p3, p4, and p5 as the probes. The EMSA revealed the shifted bands of the protein–DNA complex after the interaction between the probes of p1/p2 sequences and the LcMYB71 protein. Moreover, the signal intensity of the bound complex gradually decreased with an increase in the unlabeled competitor probes (**Figure 7C**), thus, confirming the binding of LcMYB71 to the p1 and p2 sequences.

A transient transformation assay on *N. benthamiana* leaves confirmed that LcMYB71 promoted the levels of reporters containing the p1/p2 promoters after fusion with LUC. After the co-transfection of pro1: LUC or pro2: LUC with 35S:MYB71 into *N. benthamiana*, luminescence was significantly enhanced, whereas no LUC activity was observed in NCs (pROK2, 35S:MYB71 and pro1: LUC or pro1: LUC) (**Figure 7D**). Thus, *LcMYB71* enhanced *LcNAC73* expression *in vivo*.

To determine the role of *LcMYB71* in activating *LcNAC73* by combining it with the *LcNAC73* promoter, a ChIP assay using was performed using *L. caerulea* plants overexpressing the MYB71-GFP fusion gene. Moreover, the anti-GFP antibody (ChIP+) was used for the immunoprecipitation of the ChIP products, with HA antibody-immunoprecipitated chromatin being used as the NC (ChIP−) and input as the positive control. Truncated *LcNAC73* promoters were then amplified by designing paired primers (**Figure 7E**). PCR was performed using ChIP+, ChIP−, and input as the templates. Gel electrophoresis was used to separate and visualize the ChIP-PCR products. According to the ChIP-qPCR results, two truncated promoters with specific amplification from the input control were amplified from ChIP+ but not from ChIP-control (**Figure 7F**). Similarly, the ChIP-qPCR results revealed that these two truncated promoters were significantly enriched when compared with the input (**Figure 7G**). These findings indicate that LcMYB71 combined with the *LcNAC73* promoter of *L. caerulea in vivo* and served as the upstream regulator of *LcNAC73*.

### 3.9 Expression profiles of *LcMYB71* and *LcNAC73*

To characterize the expression of *LcMYB71* and *LcNAC73* under LTS and drought stress, a qRT-PCR assay was conducted. In *L. caerulea*, *LcMYB71* and *LcNAC73* were induced by LTS and drought stress for 12 h, then downregulated to the lowest expression levels at 24 h, and gradually elevated to the peak expression levels at 48 h (**Figure 1**). Thus, the expression of *LcMYB71* and *LcNAC73* was activated under PEG and LTS. These genes are related to the response to abiotic stress. Additionally, they exhibited similar expression profiles under PEG and LTS, suggesting their involvement in the identical regulatory cascade and supporting the hypothesis that *LcMYB71* is the upstream regulator of *LcNAC73*.

The expression of *LcMYB71* and *LcNAC73* was analyzed in the OX-MYB71, KD-MYB71, and Con plants using qRT-PCR. *LcMYB71* and *LcNAC73* expression levels were significantly higher in the OX-MYB71 plants and lower in the KD-MYB71 plants than those in the Con plants (**Figure 1**). These findings indicated that *LcMYB71* activates transcription and positively modulates the expression of *LcNAC73* in *L. caerulea*.

### 3.10 Physiological analysis of *LcMYB71*

**Figure 8:**
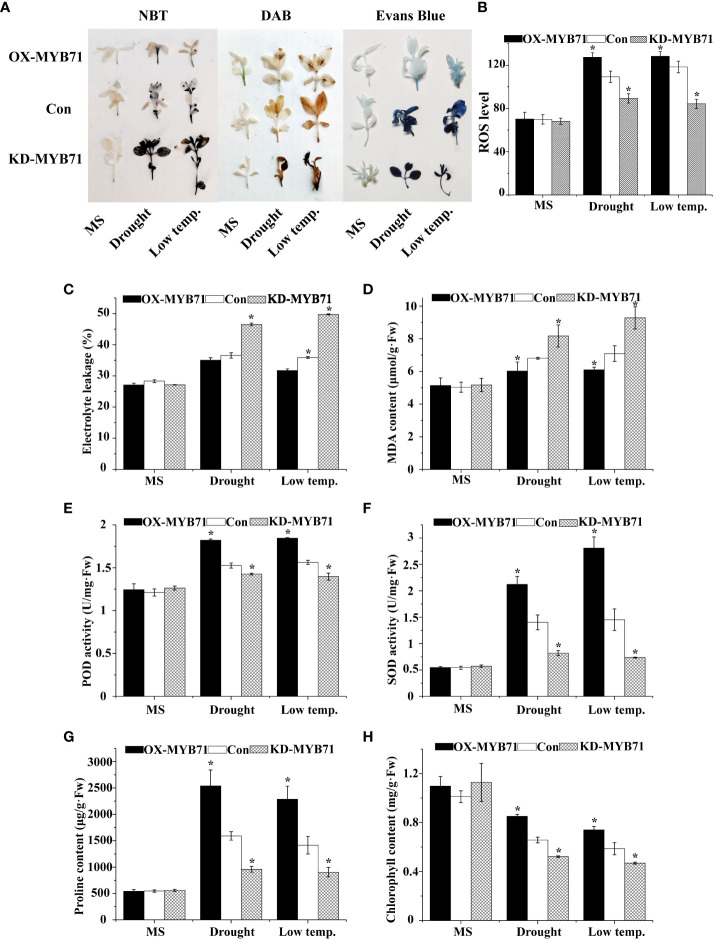
Analysis of the physiological responses mediated by *LcMYB71*. Three independent biological replications were performed. The data represent the mean values of the three independent experiments. Error bars indicate the SD. *The significant difference (t-test, P < 0.05) compared with Con. **(A)** NBT, DAB, and Evans blue staining. **(B)** ROS level. **(C)** Electrolyte leakage. **(D)** MDA content analysis. **(E)** POD activity. **(F)** SOD activity. **(G)** proline content. **(H)** chlorophyll content. OX-MYB71: *LcMYB71* transformed into *L. caerulea* for transient overexpression; Con: *L. caerulea* transiently transformed with empty pROK2 as a control; KD-MYB71: pFGC5941-MYB71 transformed into *L. caerulea* for RNAi-silencing of *LcMYB71*. 1/2MS: grown in 1/2MS medium as the normal condition control.

Under normal conditions, the O_2_^−^ and H_2_O_2_ levels were stable in the plants. However, under LTS and drought stress, their levels decreased significantly in the OX-MYB71 plants and increased in the KD -MYB71 plants compared with the Con plants (**Figure 8A**). According to the results of Evans blue staining, the staining intensities of the OX-MYB71, KD-MYB71, and Con plants were similar under normal conditions. However, after PEG or LTS treatment, membrane injury was lower in the OX-MYB71 plants and higher in the KD-MYB71 plants than in the Con plants (**Figure 8A**). Furthermore, after treatment with 20% PEG6000 or exposure to LTS, the ELR was significantly lower in the OX-MYB71 plants and significantly higher in the KD-MYB71 plants than in the Con plants (**Figure 8C**). This observation agrees with the results of Evans blue staining (**Figure 8A**). ROS level analysis and histochemical staining showed the same trend (**Figure 8B**). After treatment with 20% PEG6000 or exposure to LTS, the OX-MYB71 plants showed lower MDA levels than did the Con plants. In contrast, the KD-MYB71 plants showed a higher MDA level than did the Con plants (**Figure 8D**). Furthermore, in the OX-MYB71, KD-MYB71 and Con plants, SOD and POD activities and proline content remained stable in a normal environment with little difference. However, under 20% PEG6000 or LTS, these activities or contents increased considerably in the OX-MYB71 plants and decreased in the KD-MYB71 plants compared with those in the Con plants (**Figure 8E–G**). In the OX-MYB71, KD-MYB71, and Con plants, chlorophyll levels remained stable in a normal environment, with little difference. However, after low-temperature or drought treatment, the chlorophyll levels in OX-MYB71 plants decreased the least, followed by the Con plants, and KD-MYB71 plants showed the greatest decrease in chlorophyll levels (**Figure 8H**). Thus, *LcNAC73* enhanced the tolerance to low-temperature or drought stress.

## 4 Discussion

**Figure 9:**
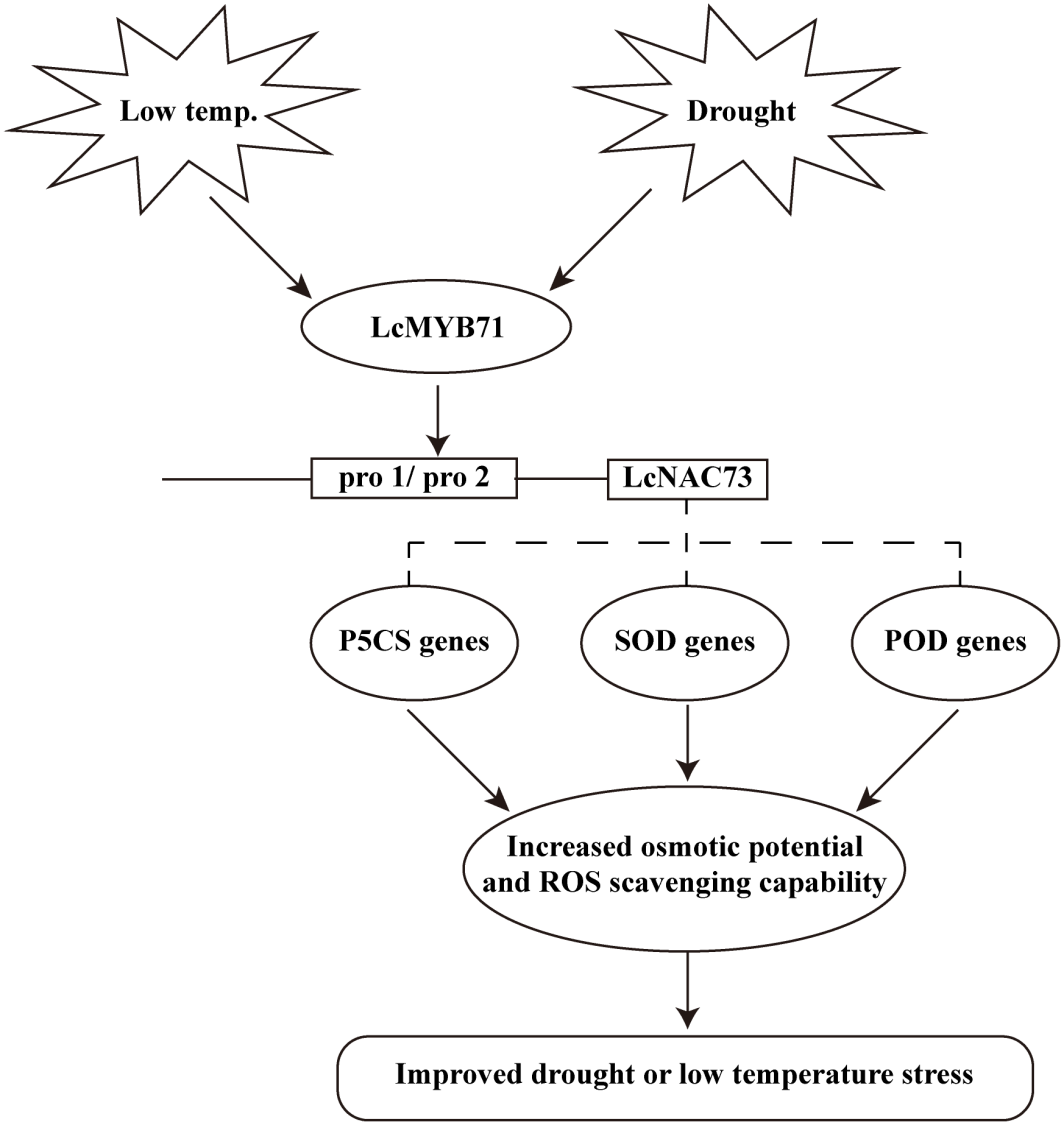
A model for *LcMYB71* and *LcNAC73* responding to low-temperature and drought stimuli. Expression of *LcMYB71* and *LcNAC73* are induced upon low-temperature and drought stress. The LcMYB71 protein then binds to the promoter of *LcNAC73* to induce ROS-related gene expression. The induction of these genes increases the osmotic potential and ROS scavenging capability. These physiological changes contribute to increased osmotic potential and enhanced ROS scavenging capability to reduce ROS damage, resulting in improved low-temperature and drought tolerance.

We found that a transcriptional cascade composed of *LcMYB71* and *LcNAC73* responded to LTS and drought stress, primarily by increasing the scavenging capacity of ROS and the proline synthesis pathway (**Figure 9**). The interaction between *LcMYB71* and *LcNAC73* was confirmed using Y1H, EMSA, and Chip-PCR (**Figure 7**). Transient transformation plants and stable transformation plants overexpressing *LcMYB71* and *LcNAC73* exhibited similar physiological changes in terms of improving tolerance to LTS and drought stress (**Figures 4**, **5**, **8**). Overexpression of the *LcMYB71* and *LcNAC73* genes in both transient and stable transformation plants reduced ELR and MDA content while increasing POD and SOD activities, proline content, chlorophyll content, ROS activity, and other physiological changes in plants. These modifications are associated with increased tolerance to LTS and drought stress.

SND 2/3/4/5 in *Arabidopsis thaliana* and *Populus trichocarpa* are reported homologs of *LcNAC73* (Zhong et al., 2021). SND 2/3/4/5 is a NAC transcription factor family nuclear protein (Fang et al., 2020). LcNAC73 is also a nuclear protein (**Figure 2**). SND1 gene expression can be significantly increased by salt and osmotic stress. SND1 directly inhibits ABA biosynthesis by inhibiting ABI4 transcripts, and SND1 overexpression improves plant stress tolerance to salt and osmotic stress (Jeong et al., 2018; Hu et al., 2019). SND1 and SND2 form a transcription cascade in poplar (hybrid T89), and drought stress can induce SND2 express. (Yu et al., 2021). *AtSND2* can promote the secondary cell wall deposition in *Arabidopsis* under LTS (Yu et al., 2021). These findings support the hypothesis that *LcNAC73* regulates the response to LTS and drought stress in *L. caerulea*.

Many transcription cascades are important in plant responses to abiotic stress. MYB108 and ANAC003 form a transcription cascade in *Arabidopsis* to change leaf senescence (Chou et al., 2018). SND1 and MYB32, for example, form a transcription cascade in poplar to respond to drought stress by inhibiting monolignol biosynthesis (Wang et al., 2011). MYBs have been identified as SND’s downstream TFs in the transcriptional regulatory networks for wood formation (McCarthy et al., 2010; Lin et al., 2013; Nakano et al., 2015; Tang et al., 2015; Jiao et al., 2019), whereas we found *LcMYB71* to be an upstream regulator of *LcNAC73* in *L. caerulea*. A transcription cascade (*LcMYB71*-*LcNAC73*) was discovered in this study. We constructed a yeast one-hybrid library containing four MYB genes (GenBank numbers: OP117115, OQ145321, OQ145322, OQ145323). In the yeast one-hybrid library, only MYB71 can interact with the promoter of *LcNAC73*, so we only focus on MYB71 in the following experiments (**Supplementary Figure 2**). These interactions may also provide new insights for the study of the stress resistance regulatory network of *L. caerulea*.

Furthermore, homologs of *LcMYB71* include *AtMYB71*/*AtMYB79*/*AtMYB121* in *A. thaliana* and MYB2 in *P. trichocarpa*. In *Arabidopsis*, the transcriptional activator MYB71 positively regulates the ABA response (Cheng et al., 2022). *MeMYB26* is a drought-responsive transcription factor in cassava related to *AtMYB71*/*AtMYB79*/*AtMYB121* (Wang et al., 2021). The findings are consistent with ours, indicating that some MYB transcription factors influence plant stress tolerance to abiotic stress and that some may share transcription pathways with *LcMYB71*.

Transient genetic transformation technology, which is suitable for plants lacking a stable transformation system, was used in this study. The plant materials used in the previous report on the *A. tumefaciens* transient genetic transformation system are *Tamarix hispida* and *Betula platyphylla* (Ji et al., 2014; Jia et al., 2022). The research object of this article was *L. caerulea*. During the process of *Agrobacterium* infection, excessive concentration of bacterial solution can cause damage to the plant; Low bacterial concentration is not conducive to the introduction of exogenous genes. At the same time, the infection time should also be strictly controlled. If the infection time is too long, it will cause the explants to suffer from hypoxia and decay due to the toxicity of *Agrobacterium*; The short duration of infection prevents successful transformation. The optimal conversion time for transient transformation of *Paeonia lactiflora* is 12 h (Guan et al., 2022). The optimal time for transient transformation of lilies (*Lilium longiflorum*) is 5 h (Wu et al., 2022). In this study, GUS staining showed the best transformation efficiency at 7h. It can be seen that different species have different requirements for bacterial infection time during the genetic transformation process. Co-culture is aimed at continuously reproducing *Agrobacterium* attached to the wound of the explants, promoting the integration of T-DNA into the plant genome. Long co-culture time can cause the explants to be poisoned and die. Song et al. found that there was no significant change in the expression of the gene after co-culturing young leaves of *Populus alba* for 3-5 days (Song et al., 2019). We found in the experiment that the transformation efficiency of *L. caerulea* was stable after co-cultivation for 3-6 days, which is similar to previous research results (Guan et al., 2022; Wu et al., 2022). The culture medium and co-transformation time used in this study differ slightly from the experimental methods used in the previous article due to the different plant materials, but the above transient genetic transformation systems achieved the goal of quickly identifying gene function and the regulatory relationship between genes.

Plant phenotypes and physiological indicators change when exposed to abiotic stresses, such as low temperature or drought, and these modifications are critical for assessing plant stress tolerance. Under adverse conditions, ROS homeostasis in plants is destroyed (Zhang et al., 2011; Mittler, 2017). By enhancing the activities of SOD, POD, and other enzymes, the ROS scavenging ability in cells is improved, cell damage is reduced, intracellular stability maintained, and the stress tolerance of plants is enhanced correspondingly (Bhattacharjee, 2010). Furthermore, under abiotic stress, the MDA content, electrolyte rate, proline content, and chlorophyll content of plants will change, so the above physiological indicators are also important factors in measuring plant stress tolerance (Amini et al., 2015; Kaur and Asthir, 2015; Ma et al., 2015; Sharma et al., 2020). The plants overexpressing *LcMYB71* and *LcNAC73* in this study improved their ability to withstand LTS and drought stress by increasing ROS scavenging capacity and proline content.

The following are some of the potential limitations of this study: (1) Four genes containing the MYB domain were connected to the pGADT7 vector separately to form the yeast one hybrid library. This library does not contain all MYB genes, so when the *LcNAC73* promoter recognized upstream regulators, it might have also combined with other MYB proteins, but this protein was not in the library we built. Thus, the upstream regulatory factors of *LcNAC73* found by us are not comprehensive. (2) The object of this study was *L. caerulea*. Currently, a stable transformation system for *L. caerulea* has not been successfully developed. To investigate gene function, we employed *A. tumefaciens* transformation technology, which may not be suitable for all plants.

Given the aforementioned constraints, the following solutions are possible: (1) Construct a plant cDNA library and use yeast one-hybrid to obtain a large number of interacting proteins; (2) conduct extensive research on *L. caerulea* tissue culture technology and develop a stable transgenic system suitable for *L. caerulea*; (3) develop a transient transformation system that can be applied to most plants and can quickly screen gene functions.

Only the molecular mechanism of the formation of the *LcMYB71*-*LcNAC73* transcription cascade was studied in depth in this study. There are numerous TFs-*LcNAC73* transcription cascades to investigate. The key research questions are as follow. What role do transcriptional cascades play in regulating plant responses to abiotic stress? Is there a transcriptional cascade that functions similarly to the *LcMYB71*–*LcNAC73*? Furthermore, what triggered the transcription cascade of *LcMYB71*-*LcNAC73* is unknown. In future work, we will investigate the issues raised above in greater depth.

## 5 Conclusions

The transcriptional cascade related to the stress tolerance to LTS and drought stress in *L. caerulea* was formed by *LcMYB71* and *LcNAC73*. The expression of *LcMYB71* and *LcNAC73* increased in response to LTS and drought stress, resulting in increased osmotic potential and ROS scavenging capacity. The above molecular and physiological changes eventually promoted tolerance to LTS and drought stress.

## Data availability statement

The datasets presented in this study can be found in online repositories. The names of the repository/repositories and accession number(s) can be found in the article/**Supplementary Material**.

## Author contributions

DZ: Funding acquisition, Writing – original draft, Writing – review & editing. YS: Software, Writing – original draft. HZ: Investigation, Writing – review & editing.

## Supplementary material

Supplementary Figure 1 Relative expression analysis of one SND gene in *L. caerulea* under low-temperature stress.

Supplementary Figure 2 Analyses of the binding of MYBs to the truncated *LcNAC73* promoter using Y1H.

Supplementary Figure 3 Under abiotic stress, plants need to take additional preventive measures and phenotypes.

Supplementary Figure 4 Phylogenetic analysis and multiple sequence alignment of *LcNAC73*.

Supplementary Figure 5 Analysis of *LcNAC73* transcript levels in overexpression (OE) lines of *Arabidopsis* plants.

Supplementary Figure 6 pCZN1-LcMYB71 were detected by SDS-Page.
