# Supplementary figures and images for: Transcriptional regulatory cascade of LcMYB71 and LcNAC73 affects low-temperature and drought stress response in Lonicera caerulea
Source: Front Plant Sci. 2023 Nov 27;14:1288947. doi: 10.3389/fpls.2023.1288947 (PMC10711284; doi:10.3389/fpls.2023.1288947)

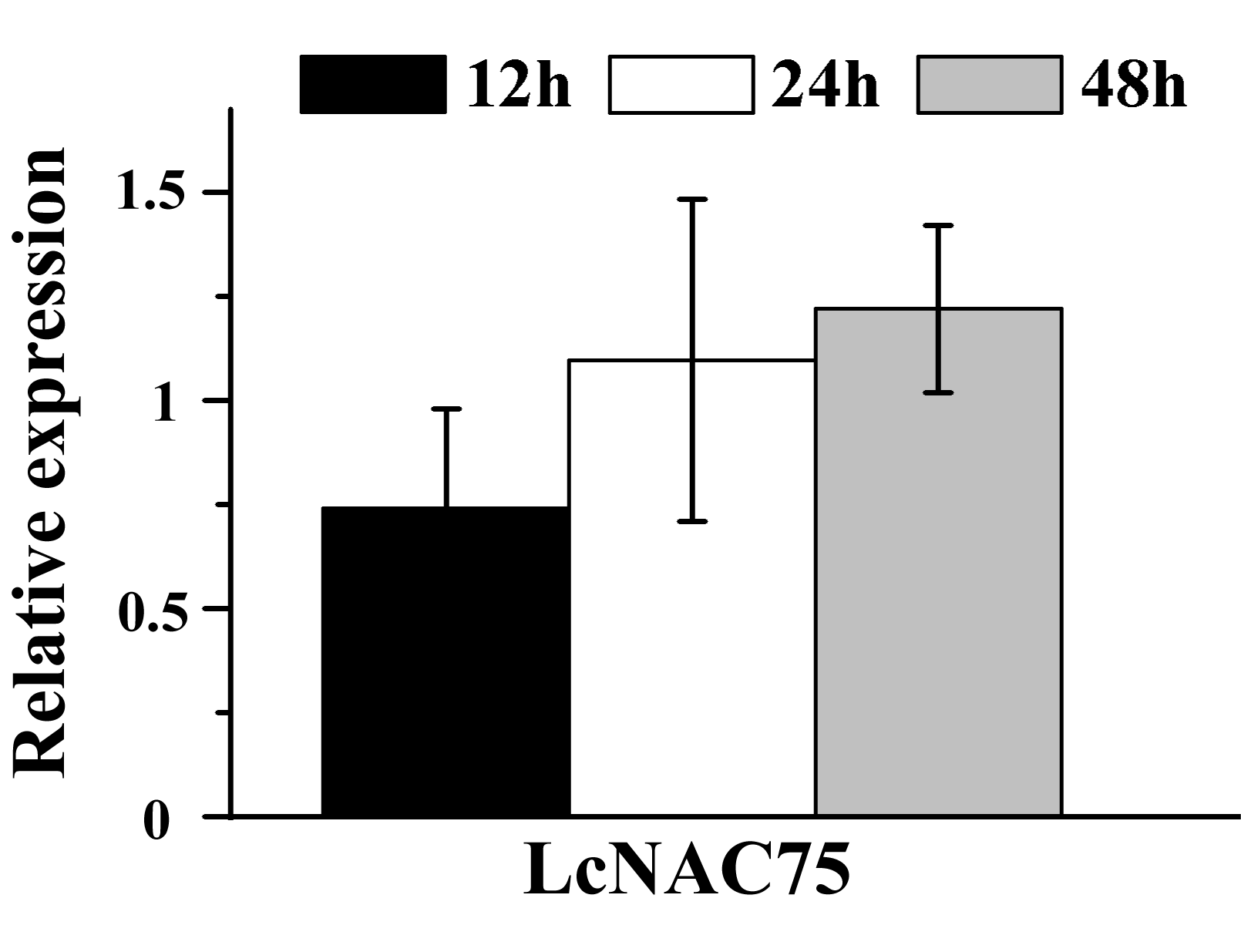

Supplement: Supplementary Figure 1 — Relative expression analysis of one SND gene in L. caerulea under low-temperature stress. [file Image_1.tif]

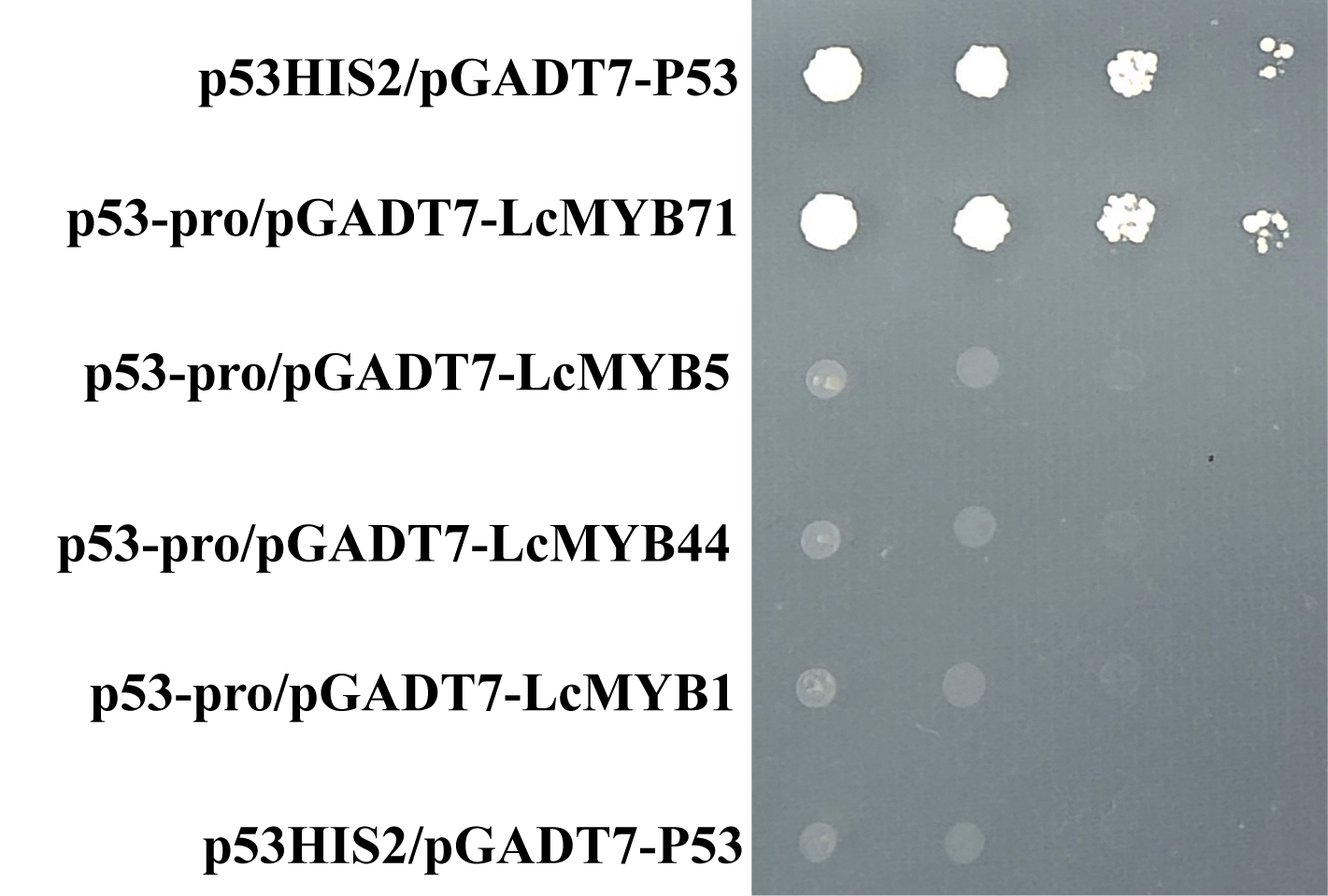

Supplement: Supplementary Figure 2 — Analyses of the binding of MYBs to the truncated LcNAC73 promoter using Y1H. [file Image_2.tif]

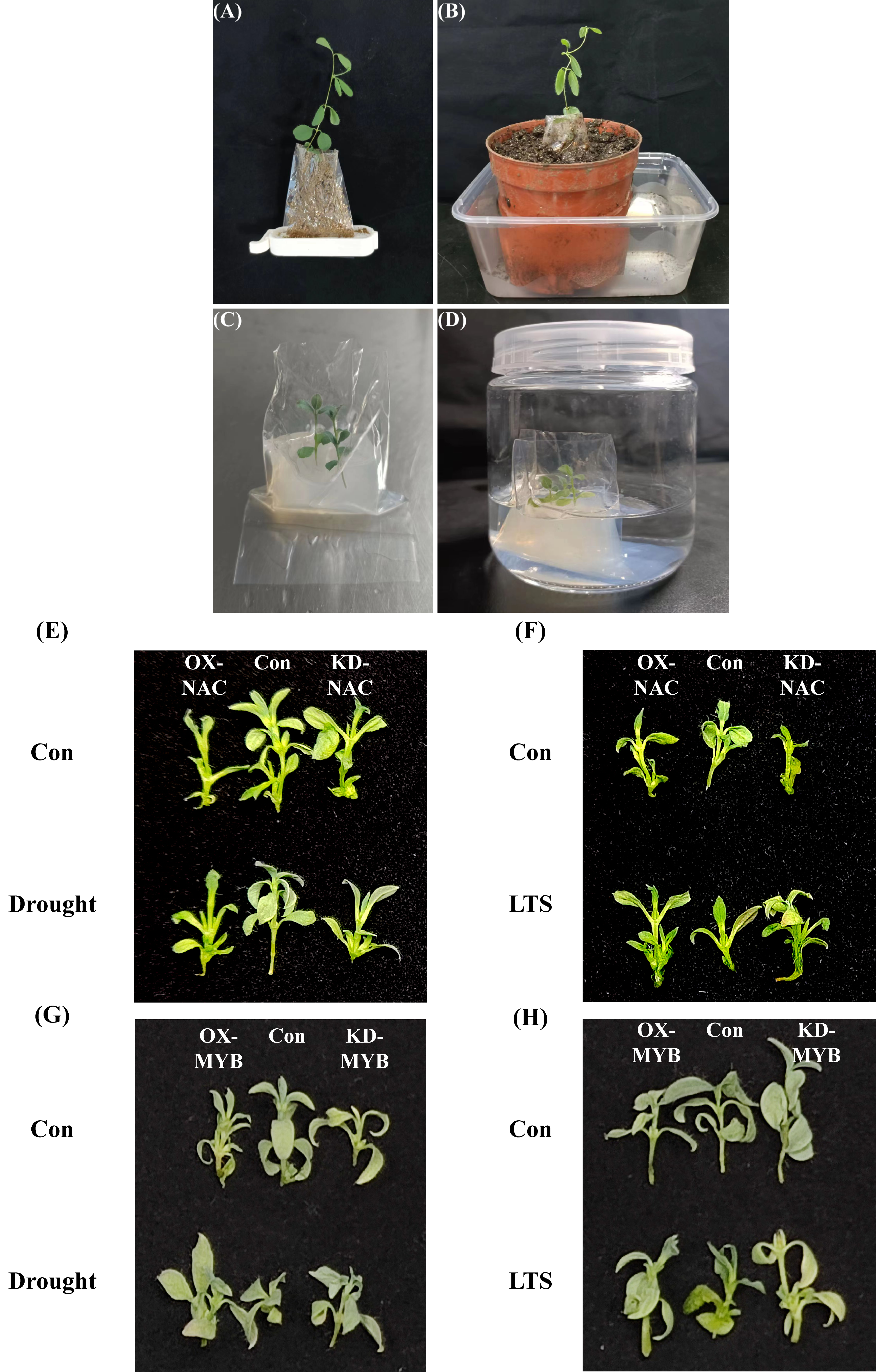

Supplement: Supplementary Figure 3 — Under abiotic stress, plants need to take additional preventive measures and phenotypes. [file Image_3.tif]

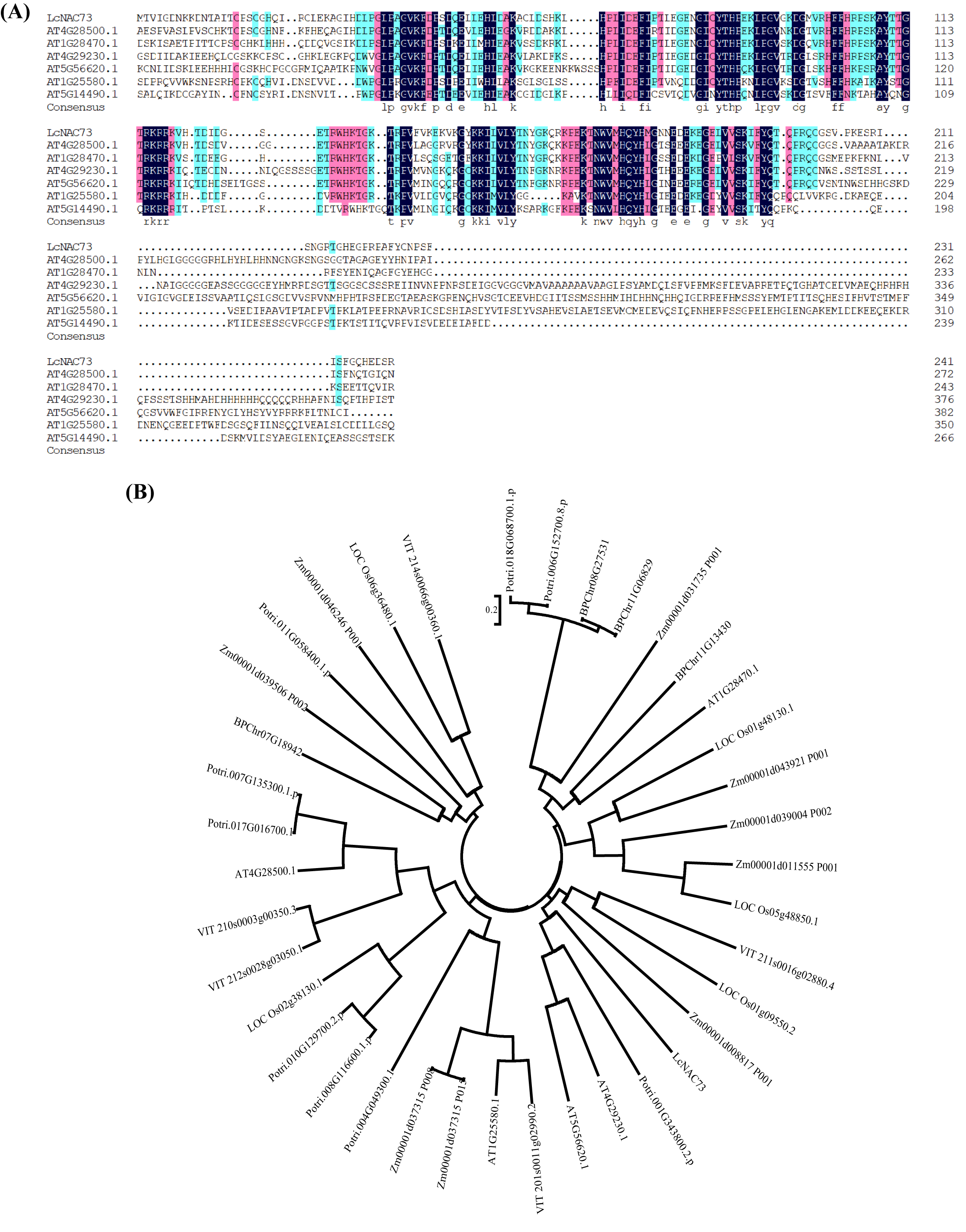

Supplement: Supplementary Figure 4 — Phylogenetic analysis and multiple sequence alignment of LcNAC73. [file Image_4.tif]

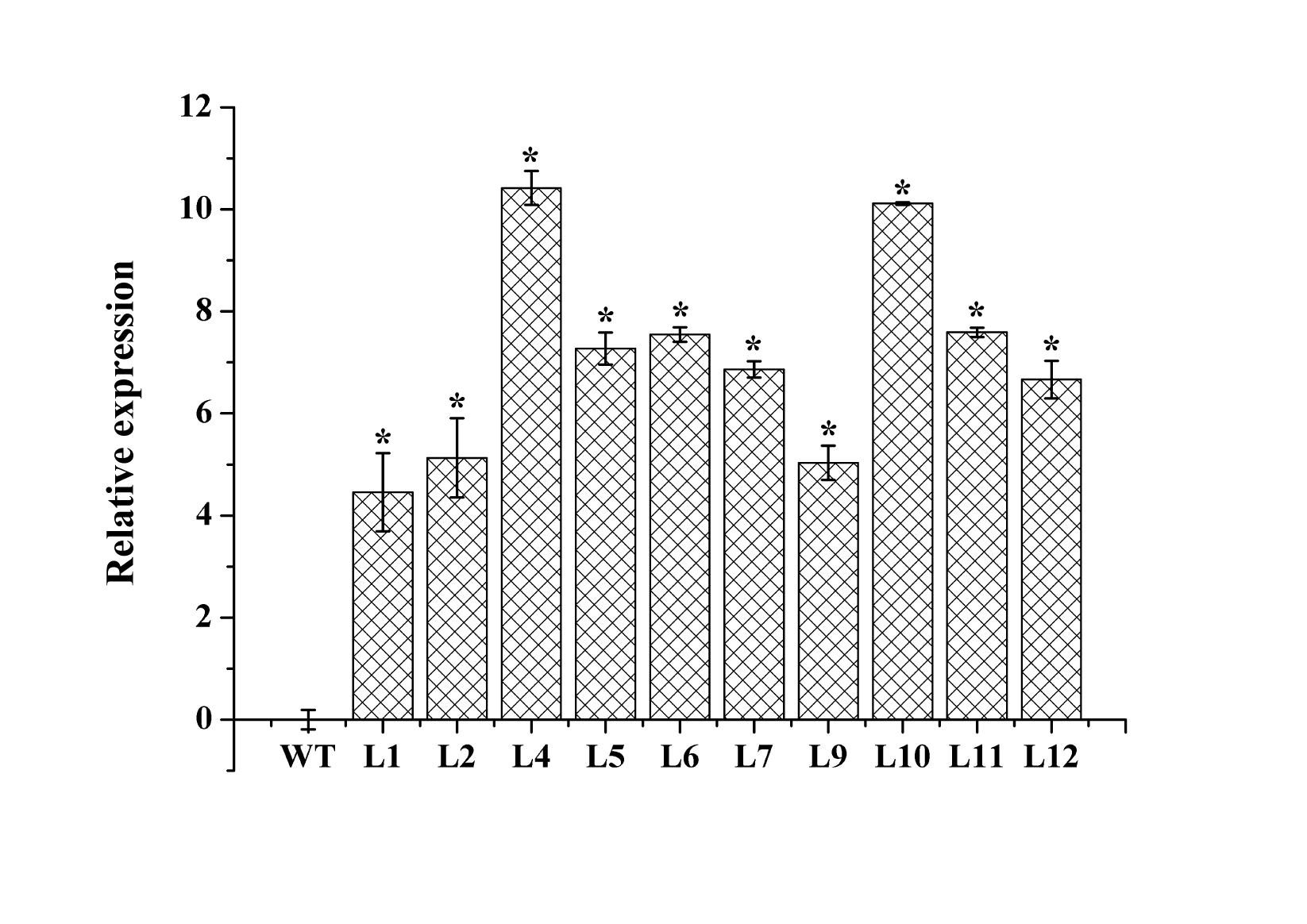

Supplement: Supplementary Figure 5 — Analysis of LcNAC73 transcript levels in overexpression (OE) lines of Arabidopsis plants. [file Image_5.tif]

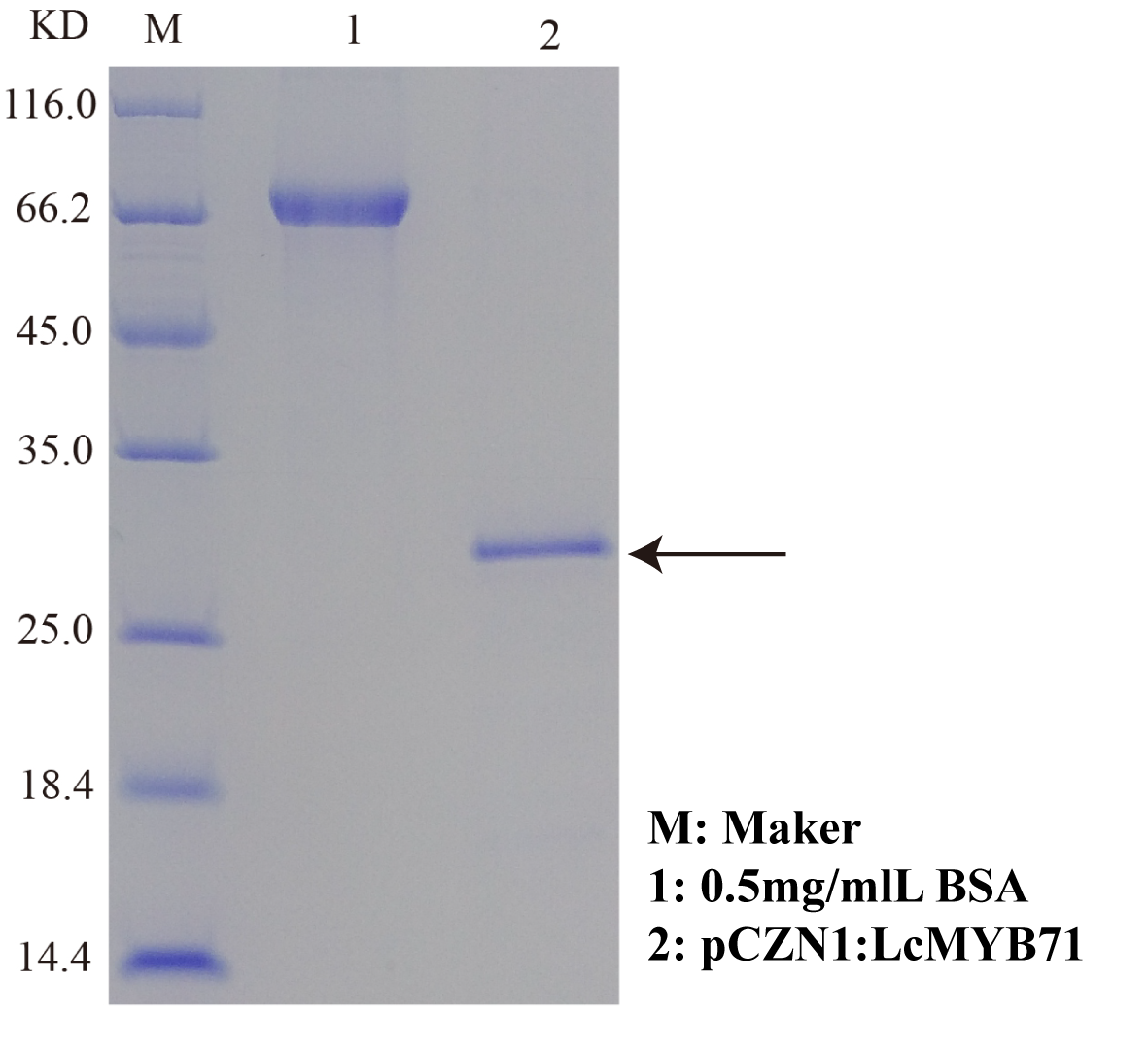

Supplement: Supplementary Figure 6 — pCZN1-LcMYB71 were detected by SDS-Page. [file Image_6.tif]
